# Transition of Neural Activity From the Chaotic Bipolar-Disorder State to the Periodic Healthy State Using External Feedback Signals

**DOI:** 10.3389/fncom.2020.00076

**Published:** 2020-08-28

**Authors:** Hirotaka Doho, Sou Nobukawa, Haruhiko Nishimura, Nobuhiko Wagatsuma, Tetsuya Takahashi

**Affiliations:** ^1^Faculty of Education, Teacher Training Division, Kochi University, Kochi, Japan; ^2^Graduate School of Applied Informatics, University of Hyogo, Kobe, Japan; ^3^Department of Computer Science, Chiba Institute of Technology, Narashino, Japan; ^4^Department of Information Science, Faculty of Science, Toho University, Funabashi, Japan; ^5^Research Center for Child Mental Development, Kanazawa University, Kanazawa, Japan; ^6^Department of Neuropsychiatry, University of Fukui, Yoshida, Japan

**Keywords:** bipolar disorder, neural network, chaotic resonance, feedback control, chaos-chaos intermittency, chronotherapy

## Abstract

Chronotherapy is a treatment for mood disorders, including major depressive disorder, mania, and bipolar disorder (BD). Neurotransmitters associated with the pathology of mood disorders exhibit circadian rhythms. A functional deficit in the neural circuits related to mood disorders disturbs the circadian rhythm; chronotherapy is an intervention that helps resynchronize the patient's biological clock with the periodic daily cycle, leading to amelioration of symptoms. In previous reports, Hadaeghi et al. proposed a non-linear dynamic model composed of the frontal and sensory cortical neural networks and the hypothalamus to explain the relationship between deficits in neural function in the frontal cortex and the disturbed circadian rhythm/mood transitions in BD (hereinafter referred to as the Hadaeghi model). In this model, neural activity in the frontal and sensory lobes exhibits periodic behavior in the healthy state; while in BD, this neural activity is in a state of chaos-chaos intermittency; this temporal departure from the healthy periodic state disturbs the circadian pacemaker in the hypothalamus. In this study, we propose an intervention based on a feedback method called the “reduced region of orbit” (RRO) method to facilitate the transition of the disturbed frontal cortical neural activity underlying BD to healthy periodic activity. Our simulation was based on the Hadaeghi model. We used an RRO feedback signal based on the return-map structure of the simulated frontal and sensory lobes to induce synchronization with a relatively weak periodic signal corresponding to the healthy condition by applying feedback of appropriate strength. The RRO feedback signal induces chaotic resonance, which facilitates the transition to healthy, periodic frontal neural activity, although this synchronization is restricted to a relatively low frequency of the periodic input signal. Additionally, applying an appropriate strength of the RRO feedback signal lowered the amplitude of the periodic input signal required to induce a synchronous state compared with the periodic signal applied alone. In conclusion, through a chaotic-resonance effect induced by the RRO feedback method, the state of the disturbed frontal neural activity characteristic of BD was transformed into a state close to healthy periodic activity by relatively weak periodic perturbations. Thus, RRO feedback-modulated chronotherapy might be an innovative new type of minimally invasive chronotherapy.

## 1. Introduction

Mood disorders, including major depressive disorder, mania, and bipolar disorder (BD), exhibit high morbidity, high suicide rates, and multiple relapses during long-term treatment; effective treatments and diagnostic methods are long-standing unmet needs (reviewed in Drevets, 2000; Kessler et al., 2003; The Wellcome Trust Case Control Consortium, 2007; Price and Drevets, 2010). Accumulating neuroimaging evidence reveals the multiple and complex pathologies of mood disorders (reviewed in Baskaran et al., 2012; Vargas et al., 2013; Chiapponi et al., 2016; Arnone, 2019). Particularly, functional magnetic resonance imaging (fMRI) and electroencephalography (EEG) have revealed region-specific enhancements and depressions in neural activity in regions such as the amygdala, hippocampus, and prefrontal cortex associated with major depressive disorder (reviewed in Baskaran et al., 2012; Arnone, 2019) and BD (Vargas et al., 2013). Furthermore, deficits in the excitatory and inhibitory neural pathways, typified as employing the neurotransmitters glutamic acid and gamma-aminobutyric acid (GABA), respectively, and abnormal cortical neural networks are reportedly also associated with mood disorders (Brambilla et al., 2003; Hasler et al., 2007; Sanacora et al., 2012; Schloesser et al., 2012; reviewed in Chiapponi et al., 2016). For the treatment of mood disorders, antidepressants (e.g., selective serotonin reuptake inhibitors, serotonin, and norepinephrine reuptake inhibitors) and mood stabilizers (e.g., lithium carbonate and clozapine) are widely used (Hirschfeld et al., 2003; López-Muñoz et al., 2006; Tobe et al., 2017). However, in the treatment of BD, mood stabilizers in particular exhibit troublesome side effects, such as progressive renal failure and a narrow therapeutic index (Hirschfeld et al., 2003; López-Muñoz et al., 2006). Therefore, alternative treatments are needed, either to relieve symptoms directly or to enhance the effects of conventional pharmacological therapy, allowing dosages to be minimized.

As an alternative treatment, chronotherapy has been garnering research attention (reviewed in Abreu and Bragança, 2015). The release of neurotransmitters associated with the pathology of mood disorders, such as serotonin, noradrenaline, glutamic acid, GABA, and dopamine, exhibits circadian rhythms (Weiner et al., 1992; Castaneda et al., 2004; Weber et al., 2004; Hampp et al., 2008; Cain et al., 2017). In mood disorders, dysregulated neural circuits disturb these circadian rhythms (Yeragani et al., 2003; Glenn et al., 2006; Bonsall et al., 2011; Moore et al., 2014; reviewed in Albrecht, 2013). Chronotherapy promotes the transition of the disturbed circadian rhythms to periodic ones, consequently leading to the improvement of symptoms (Abreu and Bragança, 2015). Chronotherapies include light therapy and combination therapy (light therapy with drugs; Leibenluft et al., 1995; Terman and Terman, 2005). However, light therapy must be individualized, and customization of the luminance and wavelength of the light for each patient is difficult. Moreover, the use of inappropriate parameters in light therapy carries a risk of inducing mixed states, hypomania, and autonomic hyperactivation in cases of BD (Terman and Terman, 2005; Sit et al., 2007; Abreu and Bragança, 2015).

Circadian rhythms are a phenomenon in which biological signals exhibiting oscillations synchronize with the daily cycle; to describe these temporal behaviors at multiple hierarchical levels, from the molecular to the synaptic network, non-linear dynamic models have been proposed (Pavlidis, 1969; Goldbeter, 1995; Kurosawa et al., 2006; reviewed in Pikovsky et al., 2003; Herzog, 2007; Pavlidis, 2012). In addition to circadian rhythms, non-linear dynamic modeling has been considered key for understanding the pathology of the transition of mood between mania and depression with a view to treatment (Daugherty et al., 2009; reviewed in Hadaeghi et al., 2013a,b). Daugherty et al. and Hadaeghi et al. have demonstrated that the mood transition is caused by the phenomenon of chaos-chaos intermittency, in which the orbit of an oscillator in the phase plane hops between separated chaotic attractor regions. Hadaeghi et al. demonstrated the effect using the forced Duffing oscillator and the Liénard oscillator (Daugherty et al., 2009; Hadaeghi et al., 2013a). Furthermore, to explain the relationships between deficits in neural networks in the frontal cortex and disturbances of circadian rhythm/mood transitions in BD, Hadaeghi et al. and Bayani et al. proposed a non-linear dynamic model (referred to as the Hadaeghi model in this study) composed of frontal and sensory cortical neural networks interacting with the hypothalamus (Hadaeghi et al., 2016; Bayani et al., 2017). In this model, activity in the neural networks of the frontal and sensory cortices exhibits periodicity in the healthy state but is transferred to a state of chaos-chaos intermittency in patients with BD. The temporal fluctuation based on the healthy periodic state reflects the controlling parameter of the circadian pacemaker in the hypothalamus (Hadaeghi et al., 2016). Consequently, disturbances in the circadian rhythm, which are observed in BD because of mood transitions, appear (Hadaeghi et al., 2016; Bayani et al., 2017). The circadian rhythms reproduced by the model are highly congruent with actual clinically observed disturbances of circadian rhythms (Hadaeghi et al., 2016).

Accumulating research on the effect of fluctuations on synchronization phenomena in non-linear systems reveals that fluctuations induce many types of synchronization, such as chaos synchronization, coherence resonance, stochastic resonance, and chaotic resonance (reviewed in Pikovsky et al., 2003; Anishchenko et al., 2007; Rajasekar and Sanjuán, 2016). The mechanism of stochastic resonance in particular, in which synchronization to a weak input signal is enhanced by additive noise, has biomedical applications, such as the development of devices and methods for enhancing human tactile sensory performance (Enders et al., 2013; Kurita et al., 2013, 2016; Seo et al., 2014). Similar to the synchronization phenomenon of stochastic resonance, in chaotic resonance, the synchronization to a weak input signal is enhanced by the internal chaotic dynamics instead of additive noise (reviewed in Anishchenko et al., 2007; Rajasekar and Sanjuán, 2016). Chaotic resonance has been widely studied in many types of systems, including neural systems (Nishimura et al., 2000; Nobukawa and Nishimura, 2016; Nobukawa et al., 2016, 2017; Baysal et al., 2019; reviewed in Nobukawa and Nishimura, 2020). In these fluctuation-enhanced synchronization phenomena, the strength of the external perturbation required for the development of a periodic state is weaker than that required for forced oscillations (Sinha, 1999; reviewed in Pikovsky et al., 2003; Anishchenko et al., 2007; Rajasekar and Sanjuán, 2016). Therefore, using these synchronization phenomena may be a strategy for administering minimally invasive chronotherapy.

According to the Hadaeghi model, in patients with BD, the presence of chaos-chaos intermittency in the neural activity of the frontal cortex disturbs the circadian rhythm (Hadaeghi et al., 2016; Bayani et al., 2017). Therefore, methods that promote the transition from chaos-chaos intermittency to periodic behavior may stabilize the disturbed circadian rhythm. As the best candidate, we proposed a chaos controlling method known as the “reduced region of orbit” (RRO) method, in which chaos-chaos intermittency is synchronized to an external, weak periodic signal using a feedback principle (Nobukawa et al., 2018b). The RRO feedback signals reduce the absolute values of local maximum and minimum values of the return-map functions, causing a bifurcation called attractor merging, which underlies the chaos-chaos intermittency (Nobukawa et al., 2018b). Because the synchronization of chaos-chaos intermittency is maximally facilitated around the attractor-merging bifurcation (review in Anishchenko et al., 2007; Rajasekar and Sanjuán, 2016), an appropriate strength of the RRO feedback signal can induce synchronization, i.e., RRO feedback signals induce chaotic resonance (Nobukawa et al., 2018b). Initially, the RRO feedback signal was applied to simple cubic map systems to induce chaotic resonance (Nobukawa et al., 2018b). Subsequently, the use of an RRO feedback signal has been applied to several types of systems, such as coupled cubic maps (Nobukawa et al., 2019a) and Chua's circuit (Nobukawa et al., 2020). These studies revealed that chaotic resonance induced by RRO feedback possesses advantages over other forms of synchronization induced by fluctuations (Nobukawa et al., 2019b, 2020). Particularly, the chaotic resonance induced by the RRO feedback method exhibits higher sensitivity than stochastic resonances induced by additive noise and are more adaptable to various types of attractor conditions (Nobukawa et al., 2019b). Studies on chaotic resonance induced by the RRO feedback method have been applied to neural systems (Nobukawa and Shibata, 2019; Nobukawa et al., 2019b). Therefore, in addition to stochastic resonance controlled by additive noise in neural systems (Enders et al., 2013; Kurita et al., 2013, 2016; Seo et al., 2014), chaotic resonance controlled by RRO feedback is at the stage where biomedical applications can be considered.

In this context, we hypothesized that the chaotic resonance induced by RRO feedback will facilitate chronotherapy by adapting to the daily neural activity of each patient, allowing for minimally invasive treatments. To verify this hypothesis, we applied chaotic resonance induced by RRO feedback signals to a model of a patient with BD based on the Hadaeghi model and evaluated the transition to periodic behavior of the disturbed frontal cortical neural activity. In detail, we first developed the RRO feedback method using the Hadaeghi model from the return-map structure of the frontal and sensory cortical neural system. Second, the chaotic resonance induced by an RRO feedback signal in combination with a weak periodic signal was evaluated. Third, the amounts of perturbation required for entrainment were compared between chaotic resonance induced by RRO feedback and synchronization induced by the application of a periodic signal alone, i.e., a forced oscillation.

## 2. Materials and Methods

### 2.1. Neural System Composed of the Frontal and Sensory Cortices

**Figure 1:**
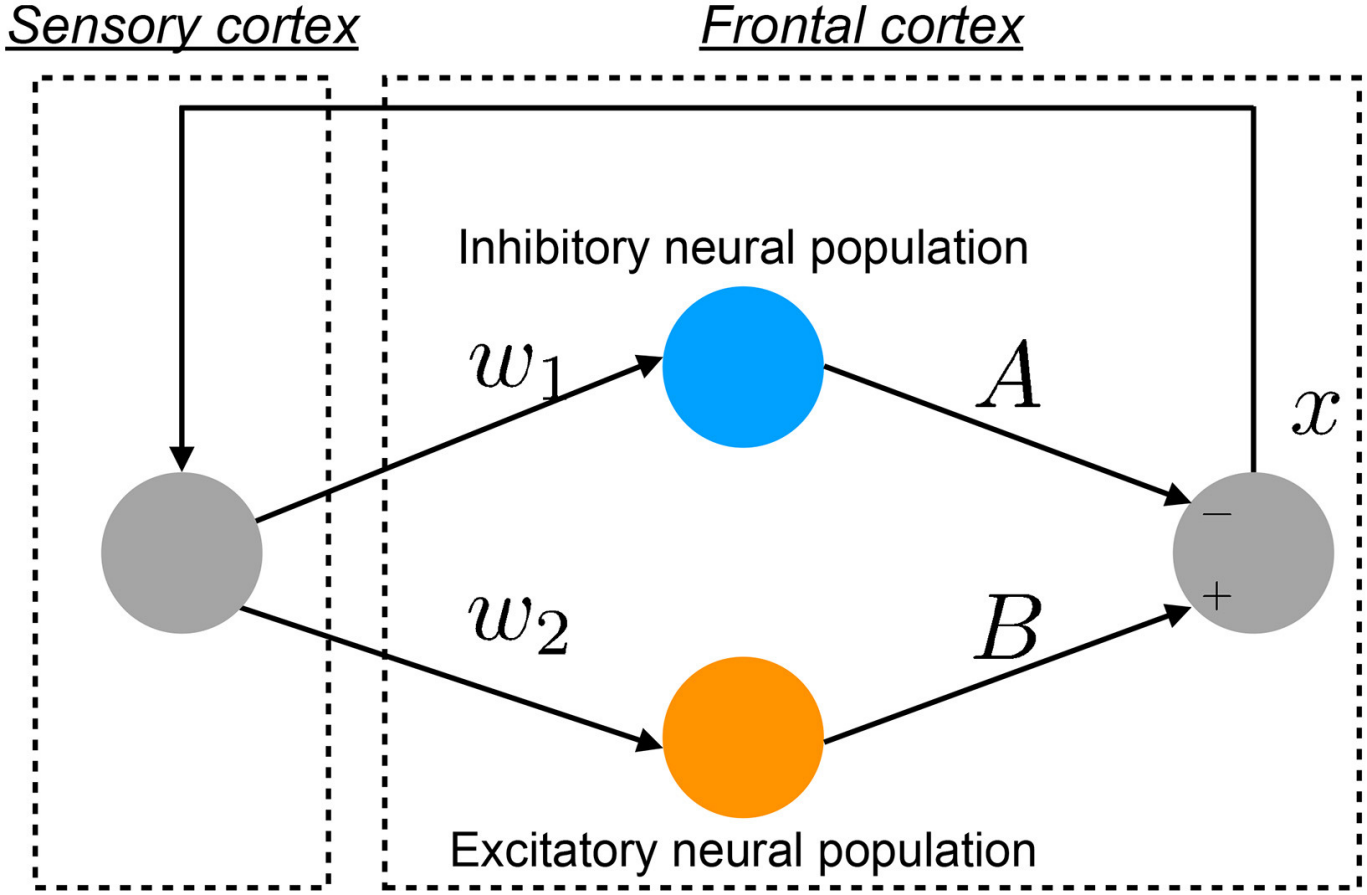
Overview of the neural system proposed by Hadaeghi et al., composed of the frontal and sensory cortices and reproducing neural activity *x* on a diurnal timescale (Hadaeghi et al., 2016).

The pathology of BD involves multiple complex neural pathways (Sanacora et al., 2012; Schloesser et al., 2012; Tobe et al., 2017). Hadaeghi et al. (2016) focused on the pathological consequences of competition between excitatory (glutamatergic) and inhibitory (GABAergic) neurons in the frontal cortex (Tretter et al., 2011; Montague et al., 2012) as major etiological factors in BD. They constructed a neural system composed of the frontal and sensory cortices to reproduce healthy and disturbed BD-associated neural activities on a diurnal timescale (Hadaeghi et al., 2016; Bayani et al., 2017). Figure 1 shows an overview of this system. This neural system has two neural pathways, excitatory and inhibitory, from the sensory cortex to the frontal cortex; the neural activity produced by the interaction between these pathways is fed back to the sensory cortex (Hadaeghi et al., 2016).

The daily neural activity of the frontal cortex *x*(*n*) (*n* = 1, 2, … days), which represents the long-term firing rate dynamics, is controlled by the competition of the excitatory and inhibitory neural populations (Hadaeghi et al., 2016):

(1) $$\begin{array}{l} x\left(n+1\right)=F\left(x\left(n\right)\right), \end{array}$$

(2) $$\begin{array}{l} F\left(x\right)=B\text{ tanh}\left(w_{2}x\right)-A\text{ tanh}\left(w_{1}x\right), \end{array}$$

where *w*_1_ and *w*_2_ are the synaptic weights of inputs to the inhibitory and excitatory neural populations, respectively. *A* and *B* correspond to the synaptic weights of the outputs of the inhibitory and excitatory neural populations, respectively, as overall neurotransmitter levels. The parameters used in this study were determined by previous research (Hadaeghi et al., 2016; Bayani et al., 2017) as follows: *w*_1_ = 0.2223, *w*_2_ = 1.487, and *B* = 5.82. In this study, as well as in the previous research, *A* is the main bifurcation parameter (Hadaeghi et al., 2016; Bayani et al., 2017).

### 2.2. Controlling Frontal Cortical Neural Activity by RRO Feedback

**Figure 2:**
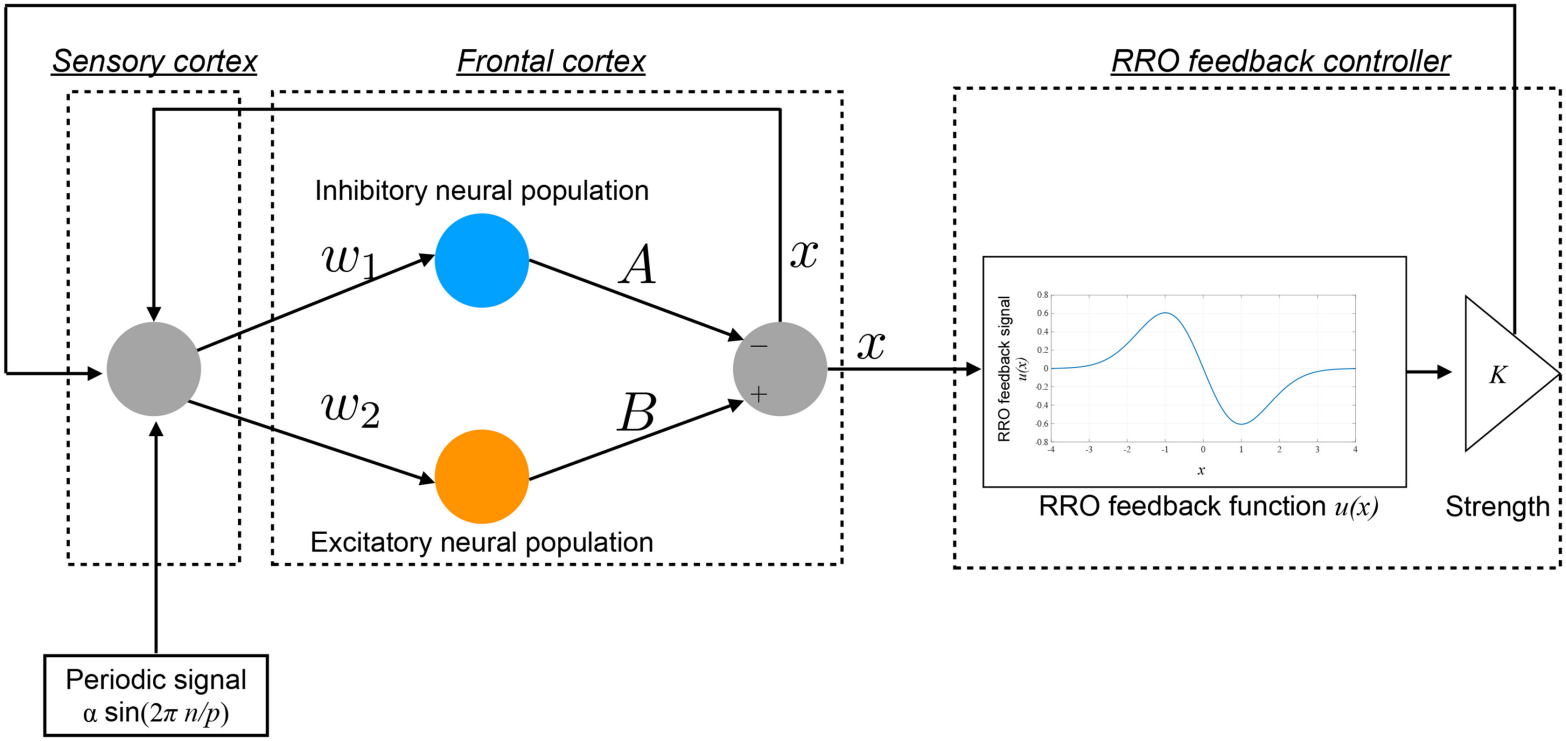
Overview of the Hadaeghi model stimulated by a reduced-region-of-orbit (RRO) feedback signal and a periodic signal.

Hadaeghi et al. demonstrated that healthy circadian rhythms and the disturbed circadian rhythms associated with BD are produced by a period-*p* state in the periodic window and a chaos-chaos intermittency state in the frontal cortical neural activity, respectively (Hadaeghi et al., 2016). The concrete behaviors of frontal neurons *x*(*n*) given by Equations (1) and (2) corresponding to healthy and BD states are demonstrated in section 3.1. In this study, we developed a feedback signal to facilitate the transition of the chaos-chaos intermittency of *x*(*n*) to the period-*p* state using an RRO-type chaotic resonance. An overview of the system for this control method is presented in Figure 2. The daily neural activity of the frontal cortex *x*(*n*) is controlled by RRO feedback signals *Ku*(*x*) and a periodic input signal *S*(*n*) = αsin(2π*n*/*p*), as follows:

(3) $$\begin{array}{l} x\left(n+1\right)=F\left(x\left(n\right)\right)+Ku\left(x\left(n\right)\right)+S\left(n\right), \end{array}$$

(4) $$\begin{array}{l} u\left(x\right)=-\left(x-x_{d}\right)\text{ exp}\left(-{\left(x-x_{d}\right)}^{2}/\left(2\sigma^{2}\right)\right). \end{array}$$

Here, *K*, *x*_*d*_, and σ represent the RRO feedback strength, the merging point of two chaotic attractors, and a parameter to determine the region of the RRO feedback effect, respectively. In this study, *x*_*d*_ = 0 and σ = 1.0 were used, because the return-map structure has a point symmetry at around *x* = 0 with local maximum and minimum values of the map function located within the region −σ < *x* < σ (σ = 1.0) (Nobukawa et al., 2018b). We used four values, 4, 8, 16, and 32, for the period *p*.

**Figure 3:**
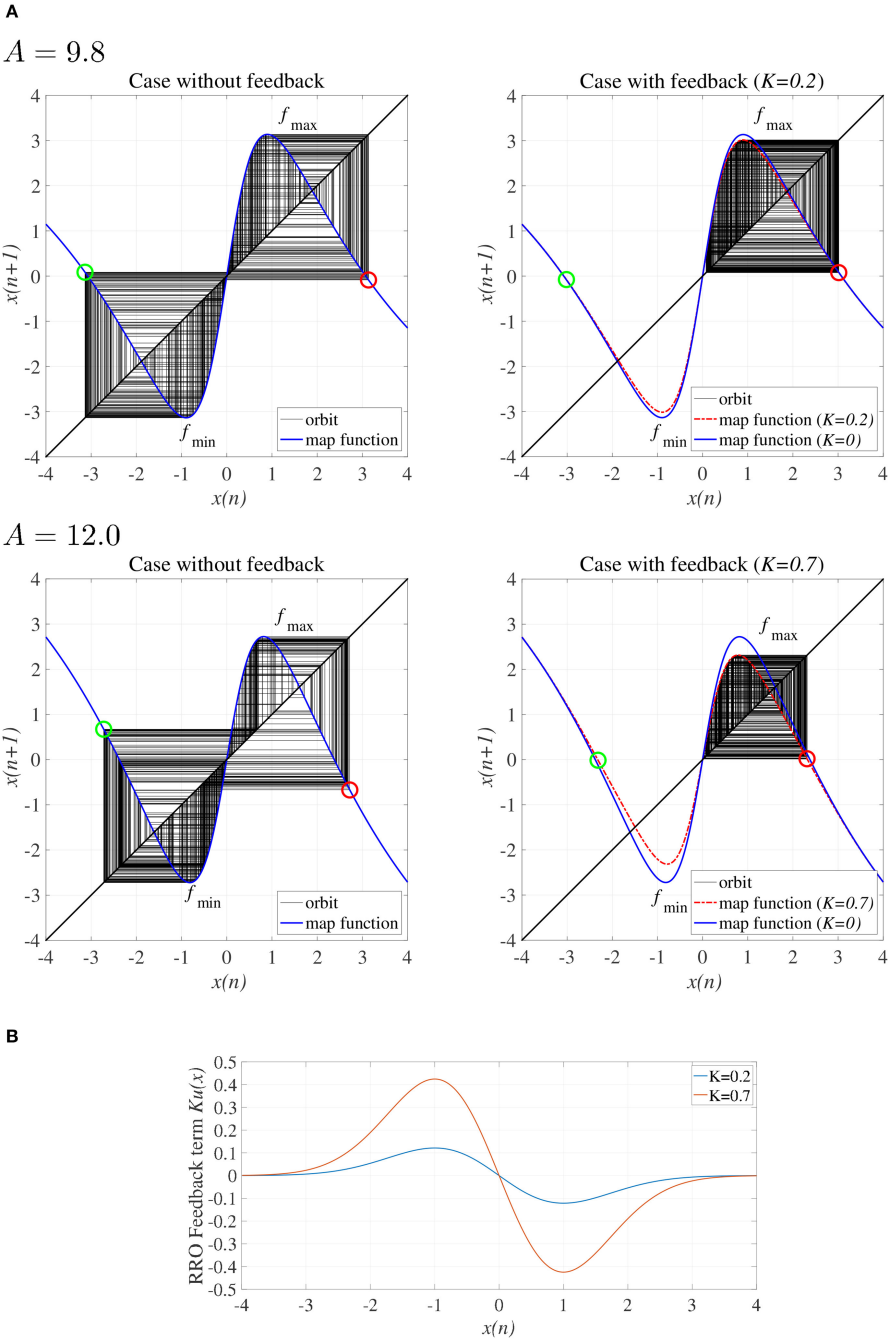
**(A)** Map function *F*(*x*) + *Ku*(*x*) for *A* = 9.8, 12.0 with and without external feedback signals in the return map between *x*(*n*) and *x*(*n* + 1). The left and right graphs indicate, respectively, map functions satisfying attractor merging conditions with *K* = 0.0 and not satisfying attractor merging conditions with *K* = 0.2 in the *A* = 9.8 case and *K* = 0.7 in the *A* = 12.0 case. Red and green circles indicate *F*(*f*_max_) + *Ku*(*f*_max_) and *F*(*f*_min_) + *Ku*(*f*_min_), respectively. RRO feedback separates the merged attractors by decreasing the absolute values of *f*_max_ and *f*_min_. **(B)** RRO feedback signal *K*(*u*(*x*)) for *K* = 0.2, 0.7. The local maximum and minimum of *K*(*u*(*x*)) are located at the local minimum and maximum of the *F* map function, respectively.

To explain the effect of the RRO feedback signal *Ku*(*x*) in the absence of the periodic input signal (α = 0), Figure 3A shows the map function of *F*(*x*) + *K*(*u*(*x*)) and the orbit *x*(*n*) in the presence/absence of RRO feedback signals. Attractor merging (chaos-chaos intermittency) occurs if *F*(*f*_max_) + *Ku*(*f*_max_) < 0 and *F*(*f*_min_) + *Ku*(*f*_min_) > 0, where *f*_max_ and *f*_min_ are the local maximum and minimum of the map function, respectively. For an inhibitory synaptic weight *A* = 9.8, 12.0 in the absence of feedback (*K* = 0), the attractor merging conditions are satisfied (left graph in Figure 3A). The orbit *x*(*n*) hops between positive and negative *x* regions, i.e., chaos-chaos intermittency arises. With positive feedback (*K* = 0.2 in the *A* = 9.8 case and *K* = 0.7 in the *A* = 12.0 case, Figure 3B), the absolute values of *f*_max_ and *f*_min_ are reduced, and the attractor merging conditions are not satisfied; the orbit *x*(*n*) is constrained to lie within either the positive or negative *x* region, depending on the initial value of *x*(0), as shown in the right graph of Figure 3A.

### 2.3. Evaluation Indices

For the evaluation of the attractor-merging bifurcation, the conditions *F*(*f*_max_) + *Ku*(*f*_max_) and *F*(*f*_min_) + *Ku*(*f*_min_) were utilized. *F*(*f*_max,min_) + *Ku*(*f*_max,min_) = 0 corresponds to the attractor-merging bifurcation point. To judge the chaotic state of frontal cortical neural activity *x*(*n*), the Lyapunov exponent was calculated as (Parker and Chua, 2012):

(5) $$\begin{array}{l} \lambda =\frac{1}{\tau M}\overset{M}{\underset{k=1}{\sum }}\text{ ln}\left(\frac{d^{k}\left(t_{l}=\tau \right)}{d^{k}\left(t_{l}=0\right)}\right). \end{array}$$

Here, $d^{k}\left(t_{l}=0\right)=d_{0}$ (*k* = 1, 2, …, *M*) denotes *M* perturbed initial conditions to *x*(*n*) applied at *n* = *n*_0_ + (*k* − 1)τ. Their time evolution for *t*_*l*_ ∈ [0:τ] is $d^{k}\left(t_{l}=\tau \right)=\left(x\left(n\right)-x^{\prime }\left(n\right)\right){|}_{n=n_{0}+k\tau }$. Furthermore, *x*′(*n*) is a perturbation applied to the orbit. λ > 0 and λ < 0 correspond to the chaotic and periodic states, respectively.

The synchronization between *x*(*n*) and *S*(*n*) was evaluated using their correlation coefficient at time delay τ as follows:

(6) $$\begin{array}{l} C\left(\tau \right)=\frac{C_{sx}\left(\tau \right)}{\sqrt{C_{ss}C_{xx}}}, \end{array}$$

(7) $$\begin{array}{l} C_{sx}\left(\tau \right)=\langle \left(S\left(n+\tau \right)-\langle S\rangle \right)\left(x\left(n\right)-\langle x\rangle \right)\rangle , \end{array}$$

(8) $$\begin{array}{l} C_{ss}=\langle {\left(S\left(n\right)-\langle S\rangle \right)}^{2}\rangle , \end{array}$$

(9) $$\begin{array}{l} C_{xx}=\langle {\left(x\left(n\right)-\langle x\rangle \right)}^{2}\rangle , \end{array}$$

where 〈·〉 denotes the average in *n*. In this study, τ is set to the value for $\underset{\tau }{\max}C\left(\tau \right)$ in each time series of *x*(*n*). The values for $\underset{\tau }{\max}C\left(\tau \right)$ are measured against ten trials with different initial values of *x*(0).

To evaluate the amount of the perturbation due to the RRO feedback signal *Ku*(*x*) plus the periodic input signal *S*(*n*), we used the temporal mean value of the squared perturbations:

(10) $$\Xi =\langle {\left(Ku(x(n))\right)}^{2}+{\left(S(n)\right)}^{2}\rangle ,$$

where 〈·〉 denotes the average in *n*. The values for Ξ are measured against ten trials with different initial values of *x*(0).

## 3. Results

### 3.1. Frontal Cortical Neural Activity on a Diurnal Timescale

**Figure 4:**
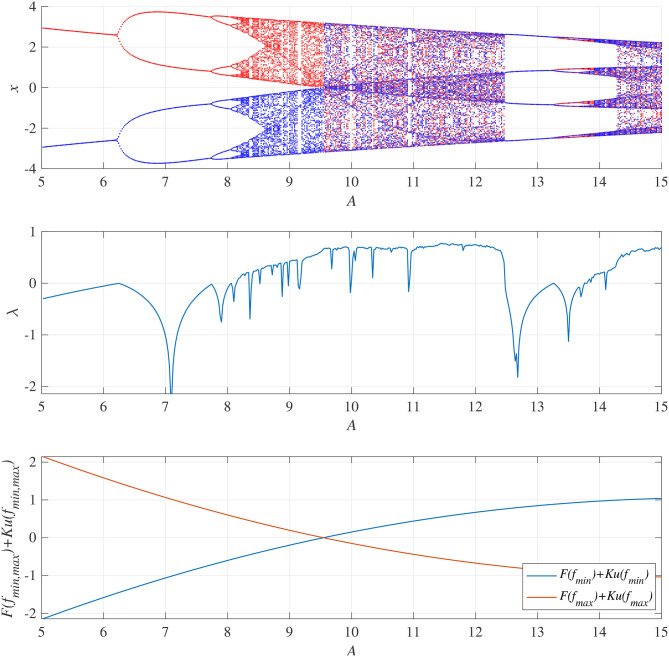
System behaviors in the neural network comprised the frontal and sensory cortices as a function of the synaptic weight from the inhibitory neural population, *A*, in the absence of feedback and periodic signals (*K* = 0, α = 0). **(Top)** Bifurcation diagram of the frontal neural activity *x*(*n*) represented by Equation (1) as a function of *A*. Blue and red dots indicate positive and negative initial values of *x*(0), respectively. **(Middle)** Lyapunov exponent λ as a function of *A*. **(Bottom)** *F*(*f*_min,max_) + *Ku*(*f*_min,max_) as a function of *A*. The frontal neural behavior in the periodic window 12.5 ≲ *A* ≲ 13.5 corresponds to that of healthy controls (HC), while the chaos-chaos intermittent behavior in 9.8 ≲ *A* ≲ 12.5 and *A* ≳ 13.5 corresponds to that of patients with BD (Hadaeghi et al., 2016).

**Figure 5:**
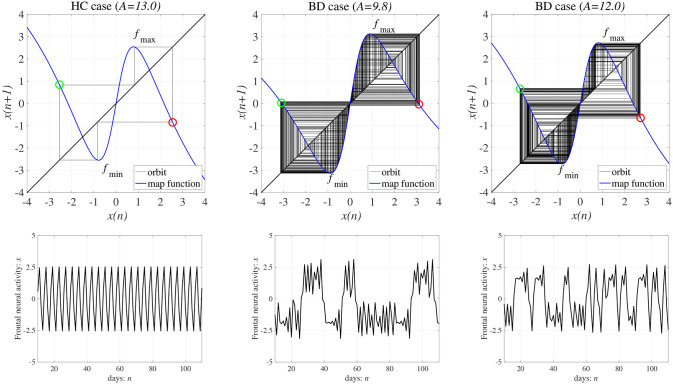
**(Upper)** Map function *F*(*x*) (the orbit in the return map) and **(Lower)** a time series showing frontal cortical neural activity *x*(*n*) in the absence of external feedback or periodic input signals (*K* = 0, α = 0). (left) Healthy control (HC) (weight from the inhibitory neural population *A* = 13.0) and (middle and right) bipolar disorder (BD) (*A* = 9.8, 12.0). In the return maps, the red and green circles indicate *F*(*f*_max_) + *Ku*(*f*_max_) and *F*(*f*_min_) + *Ku*(*f*_min_), respectively. In both HC and BD, the attractor merging condition is satisfied with *F*(*f*_max_) + *Ku*(*f*_max_) < 0 and *F*(*f*_min_) + *Ku*(*f*_min_) > 0; the periodic and chaos-chaos intermittent states correspond to HC and BD, respectively.

We demonstrated activity in a neural system composed of the frontal and sensory cortices. Figure 4 shows the bifurcation diagram of the frontal neural activity *x*(*n*), Lyapunov exponent λ, and *F*(*f*_min,max_) + *Ku*(*f*_min,max_) as functions of synaptic weights from the inhibitory neural population *A* in the absence of a feedback or periodic signal (*K* = 0, α = 0). With an increase in the *A* value, *x*(*n*) exhibits a period-doubling bifurcation and enters a chaotic state *A* ≳ 8.1 (λ > 0). In 8.1 ≲ *A* ≲ 9.8, *x*(*n*) is trapped in either the negative or the positive region, depending on the initial values of *x*(0), *F*(*f*_min_) + *Ku*(*f*_min_) < 0, and *F*(*f*_max_) + *Ku*(*f*_max_) > 0. The attractor merging conditions *F*(*f*_min_) + *Ku*(*f*_min_) > 0 and *F*(*f*_max_) + *Ku*(*f*_max_) < 0 are satisfied in *A* ≳ 9.8; consequently, *x*(*n*) hops back and forth between negative and positive regions, which is known as chaos-chaos intermittency. This effect corresponds with the merger of attractors in the negative and positive regions of the bifurcation diagram. The window of periodicity is 12.5 ≲ *A* ≲ 13.5. Hadaeghi et al. considered that frontal neural activity in the periodic window corresponds to that of healthy subjects (healthy control [HC]), whereas chaos-chaos intermittent activity corresponds to that of patients with BD (Hadaeghi et al., 2016). Figure 5 shows typical examples of the frontal neural activity *x*(*n*) governed by Equation (1) in HCs and in patients with BD. At *A* = 13.0, corresponding with typical HC behavior, *x*(*n*) exhibits the periodic-4 state, where the parameter set is located in the periodic window in the top part of Figure 4. In this periodic window, various period-*p* states exist through period-doubling bifurcation; therefore, as healthy period-*p* states, we used *p* = 4, 8, 16, 32 in this study. At *A* = 9.8, 12.0, corresponding to BD behavior, *x*(*n*) exhibits chaos-chaos intermittency. In both HC and BD cases, the attractor merging condition is satisfied with *F*(*f*_max_) + *Ku*(*f*_max_) < 0 and *F*(*f*_min_) + *Ku*(*f*_min_) > 0.

### 3.2. Transition From Disturbed Neural Activity to a Periodic State by RRO Feedback Plus Periodic Input Signal

**Figure 6:**
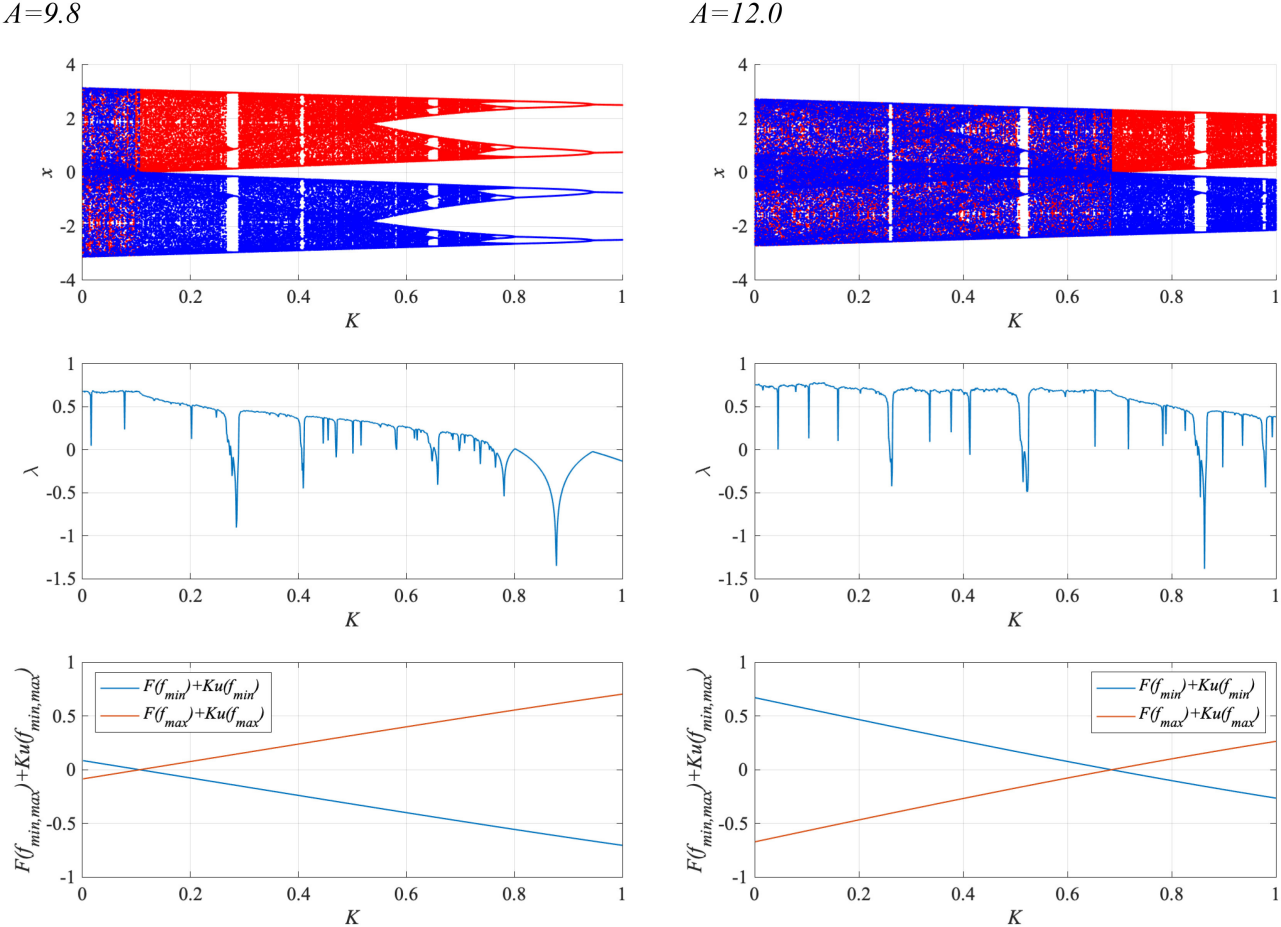
System behaviors in the neural net comprised of the frontal and sensory cortices as a function of the RRO feedback strength *K*, in the absence of a periodic signal (α = 0), for BD (*A* = 9.8, 12.0). **(Top)** Bifurcation diagram of the frontal neural activity *x*(*n*) represented by Equation (3) as a function of *K*. Blue and red dots indicate positive and negative initial values of *x*(0), respectively. **(Middle)** Lyapunov exponent λ as a function of *K*. **(Bottom)** *F*(*f*_min,max_) + *Ku*(*f*_min,max_) as functions of *K*. The positive and negative regions of the merged chaotic attractor (λ > 0) were separated by breaking the attractor merging conditions: *F*(*f*_min_) + *Ku*(*f*_min_) > 0 and *F*(*f*_max_) + *Ku*(*f*_max_) < 0 in *K* ≳ 0.1 for the *A* = 9.8 case and in *K* ≳ 0.7 for the *A* = 12.0 case.

To enhance synchronization to weak input signals, the system parameters must be adjusted to those of the attractor-merging bifurcation (Nobukawa et al., 2018b). Figure 6 shows the behavior of the neural system composed of the frontal and sensory cortices as a function of RRO feedback strength *K*, in the absence of a periodic signal (α = 0), for BD (*A* = 9.8, 12.0). Shown are the bifurcation diagram of the frontal neural activity *x*(*n*) given by Equation (3), the Lyapunov exponent λ, and *F*(*f*_min,max_) + *Ku*(*f*_min,max_). The separation of merged chaotic attractors (λ > 0) into positive and negative regions arises at the region for *F*(*f*_min_) + *Ku*(*f*_min_) < 0, *F*(*f*_max_) + *Ku*(*f*_max_) > 0 in *K* ≳ 0.1 for the *A* = 9.8 case and in *K* ≳ 0.7 for the *A* = 12.0 case.

**Figure 7:**
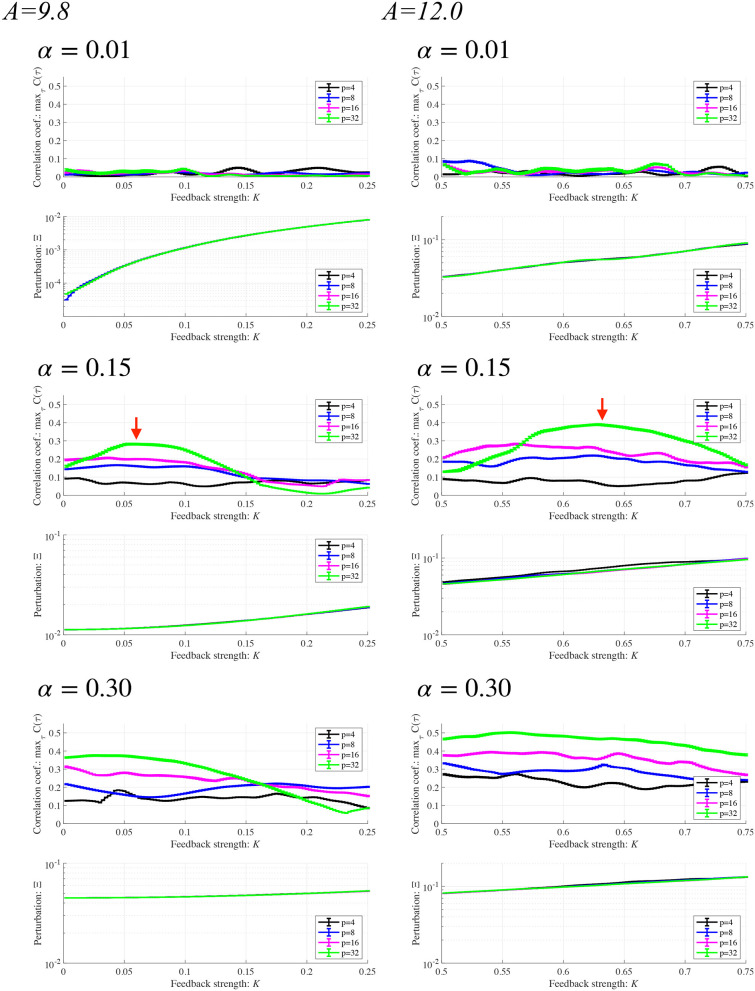
Synchronization of neural activity *x*(*n*) to a weak periodic input signal *S*(*n*) (α = 0.01, 0.15, 0.3 and *p* = 4, 8, 16, 32) and perturbations of the RRO feedback signal and the periodic input signal in BD cases (*A* = 9.8, 12.0). Here, the period *p* values are chosen based on the healthy periodic-*p* states locating in the periodic window in 12.5 ≲ *A* ≲ 13.5 in Figure 4. Dependence of **(Upper)** $\underset{\tau }{\max}C\left(\tau \right)$ and **(Lower)** Ξ on the RRO feedback strength *K*. Solid black lines and error bars show the mean and standard deviation across ten trials. In the lower panels, the scales of the vertical axes differ. In the case with α = 0.15 and *p* = 32 (represented by red arrows), $\underset{\tau }{\max}C\left(\tau \right)$ exhibits a unimodal maximum ($\underset{\tau }{\max}C\left(\tau \right)\approx 0.3$ in *A* = 9.8 and $\underset{\tau }{\max}C\left(\tau \right)\approx 0.4$ in *A* = 12.0) at around the attractor-merging bifurcation at *K*≈0.06 in *A* = 9.8, and *K*≈0.63 in *A* = 12.0. At this *K* condition, the perturbation amounts Ξ are 0.012, 0.049 in the *A* = 9.8, 12.0 cases, respectively.

**Figure 8:**
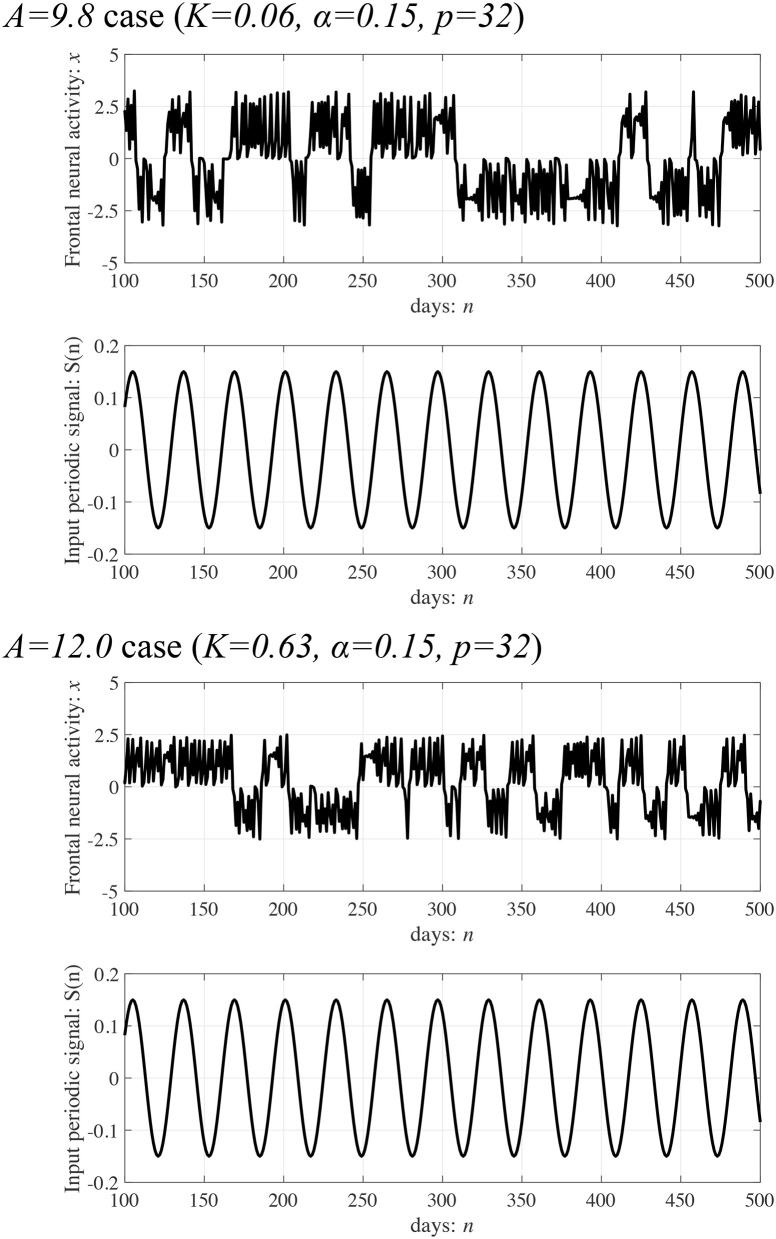
Typical time series of frontal neural activity *x*(*n*) in synchronization with a weak periodic input signal *S*(*n*) under the conditions for inducing chaotic resonance shown in Figure 7. Synchronization between the chaos-chaos intermittency of *x*(*n*) and the periodic input signal *S*(*n*) is shown, which features hopping between positive and negative *x*(*n*) regions ($\underset{\tau }{\max}C\left(\tau \right)\approx 0.3,0.4$ in *A* = 9.8, 12.0 cases, respectively).

**Figure 9:**
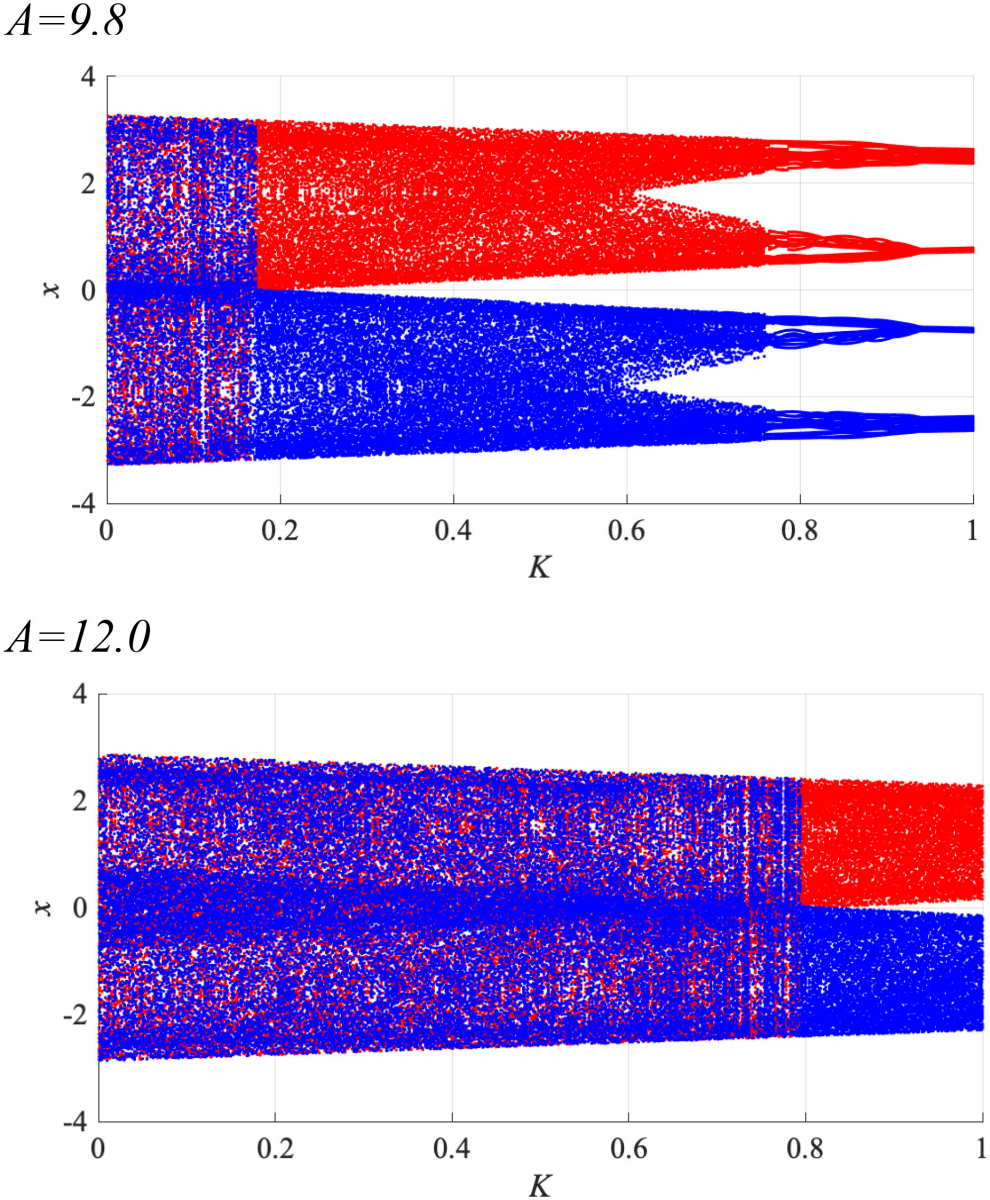
Bifurcation diagram of the frontal neural activity *x*(*n*) represented by Equation (3) as a function of RRO feedback strength *K* under a weak periodic input signal *S*(*n*) (*p* = 32, α = 0.15) in BD (*A* = 9.8, 12.0) cases. Blue and red dots indicate positive and negative initial values of *x*(0), respectively. The chaos-chaos intermittency between positive and negative *x*(*n*) regions is maintained in *K* ≲ 0.18 for the *A* = 9.8 case and in *K* ≲ 0.79 for the *A* = 12.0 case.

Subsequently, synchronization of *x*(*n*) to a weak periodic input signal *S*(*n*) (α = 0.01, 0.15, 0.3 and *p* = 4, 8, 16, 32) and the evaluated perturbations of the RRO feedback and periodic signals are shown. Here, the *p* values are chosen based on the healthy periodic-*p* states locating at the periodic window in 12.5 ≲ *A* ≲ 13.5. Figure 7 shows the dependence of $\underset{\tau }{\max}C\left(\tau \right)$ and Ξ on the RRO feedback strength *K*. In the case with α = 0.15 and *p* = 32, $\underset{\tau }{\max}C\left(\tau \right)$ exhibits a unimodal maximum ($\underset{\tau }{\max}C\left(\tau \right)\approx 0.3$ in *A* = 9.8 and $\underset{\tau }{\max}C\left(\tau \right)\approx 0.4$ in *A* = 12.0) at around the attractor-merging bifurcation defined as *F*(*f*_min,max_) + *Ku*(*f*_min,max_) = 0 at *K* ≈ 0.06 in *A* = 9.8 and *K*≈0.63 in *A* = 12.0 (see Figure 6), i.e., chaotic resonance is induced by the RRO feedback signal. Therefore, applying the RRO feedback signal together with a weak periodic signal brings the neural activity *x*(*n*) of BD close to the healthy periodic state. This chaotic resonance is induced when the perturbation Ξ = 0.012, 0.049, at *A* = 9.8, 12.0, respectively. This perturbation is relatively small in comparison to the variation range: −2.5 ≲ *x*(*n*) ≲ 2.5, as shown in the bifurcation diagram of Figure 6. Under conditions of higher input frequency (*p* = 2, 4, 8, 16) or weaker signal strength (α = 0.01), the values of $\underset{\tau }{\max}C\left(\tau \right)$ are significantly reduced. At stronger signal strength (α = 0.3), the values of $\underset{\tau }{\max}C\left(\tau \right)$ exhibit a tendency to decrease monotonically with increasing *K*. Thus, chaotic resonance can be induced by RRO feedback signals at an appropriate signal strength and frequency. Figure 8 shows a typical time series of frontal neural activity *x*(*n*) in synchronization with a weak periodic input signal *S*(*n*) under conditions that induce chaotic resonance in Figure 7, i.e., *p* = 32, α = 0.15, and *K* = 0.06 in the *A* = 9.8 case; and *K* = 0.63 in the *A* = 12.0 case. The result shows synchronization between the chaos-chaos intermittency of *x*(*n*) and the periodic input signal *S*(*n*), with hopping between positive and negative *x*(*n*) regions. Additionally, Figure 9 shows the bifurcation diagram of *x*(*n*) represented by Equation (3) as a function of *K* under a weak periodic input signal *S*(*n*) (*p* = 32, α = 0.15) in BD, in (*A* = 9.8, 12.0) cases. The chaos-chaos intermittency between positive and negative *x*(*n*) regions is maintained until around the peak of $\underset{\tau }{\max}C\left(\tau \right)$ (represented in Figure 7) in *K* ≲ 0.18 for the *A* = 9.8 case and in *K* ≲ 0.79 for the *A* = 12.0 case. Therefore, the chaotic resonance confirmed in Figure 7 produces synchronization of the chaos-chaos intermittency with the periodic input signal *S*(*n*).

**Figure 10:**
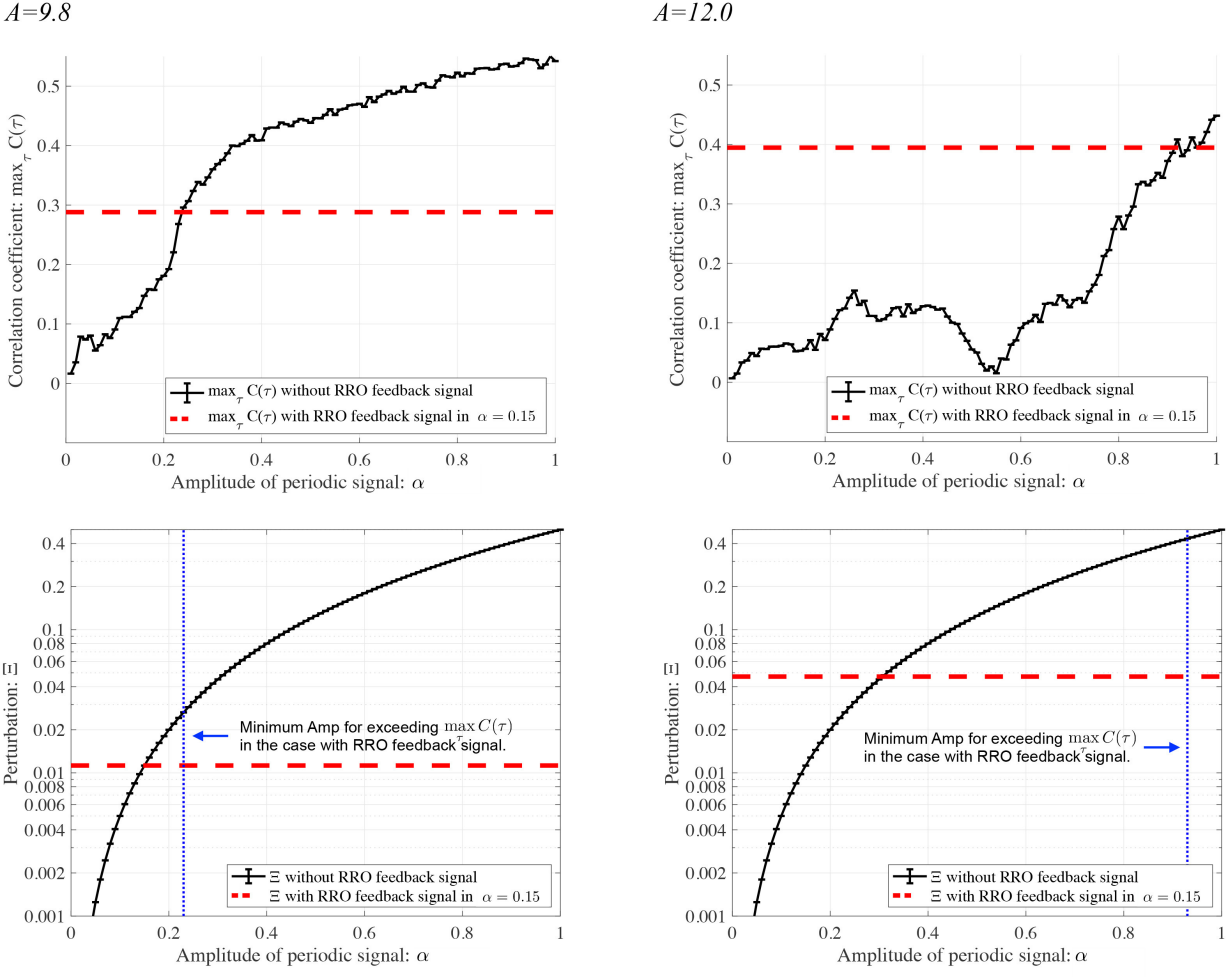
Synchronization of neural activity *x*(*n*) to a periodic input signal *S*(*n*) (*p* = 32) and perturbation by this signal in the absence of RRO feedback. Dependence of **(Upper)** $\underset{\tau }{\max}C\left(\tau \right)$ and **(Lower)** Ξ on signal strength α. Solid black lines and error bars show the mean and standard deviation across ten trials. Horizontal red dashed lines in the upper figure indicate the maximum values of $\underset{\tau }{\max}C\left(\tau \right)$ observed for the RRO feedback signals shown in Figure 7. Vertical blue dotted lines in the lower figures give the minimum signal strengths α needed for exceeding the maximum value of $\underset{\tau }{\max}C\left(\tau \right)$ in the presence of the RRO feedback signals given in Figure 7. Horizontal red dashed lines in the lower figures indicate the values of Ξ at *K*, where $\underset{\tau }{\max}C\left(\tau \right)$ peaks in Figure 7. Compared with runs having an RRO feedback signal, a larger perturbation (Ξ ≳ 0.025 in the *A* = 9.8 case and Ξ ≳ 0.41 in the *A* = 12.0 case) is needed to achieve the synchronous state with $\underset{\tau }{\max}C\left(\tau \right)≳0.3$ in the *A* = 9.8 case and $\underset{\tau }{\max}C\left(\tau \right)≳0.4$ in the *A* = 12.0 case.

To evaluate the effect of the RRO feedback signal on synchronization, we compared the synchronization induced by RRO feedback with that in its absence (*K* = 0). Figure 10 shows the dependence of $\underset{\tau }{\max}C\left(\tau \right)$ and Ξ on the signal strength α in the case of no RRO feedback under the condition where chaotic resonance is induced by the RRO feedback signal at *p* = 32 in Figure 7. In the *A* = 9.8 case with α ≳ 0.22, $\underset{\tau }{\max}C\left(\tau \right)$ exceeds 0.3, which corresponds to the maximum value of $\underset{\tau }{\max}C\left(\tau \right)$ under RRO feedback presented in Figure 7. Moreover, the perturbation amount Ξ at α≈0.22 required for accomplishing $\underset{\tau }{\max}C\left(\tau \right)\approx 0.3$ is approximately 0.025; however, under RRO feedback, it is Ξ≈0.012 at *K*≈0.06 for a peak correlation of $\underset{\tau }{\max}C\left(\tau \right)\approx 0.3$. Therefore, the RRO feedback signal reduces the amount of perturbation needed for the transition to the periodic state. In the *A* = 12.0 case, the same tendency seen in the *A* = 9.8 case is confirmed. That is, the perturbation amount Ξ at α≈0.95 required for accomplishing $\underset{\tau }{\max}C\left(\tau \right)\approx 0.4$ is approximately 0.41; however, under RRO feedback, it is Ξ≈0.049 at *K*≈0.63 for a peak correlation of $\underset{\tau }{\max}C\left(\tau \right)\approx \;0.4$.

## 4. Discussion and Conclusions

In this study, we verified our hypothesis that chaotic resonance induced by RRO feedback signals can enable the delivery of chronotherapy by minimally invasive treatments. In a simulation based on the Hadaeghi model, we evaluated the transition of the disturbed frontal cortical neural activity corresponding to BD to the periodic behavior found in the HCs that was induced by RRO feedback signals. We found that the RRO feedback signal, which is based on the return-map structure of the modeled frontal and sensory cortical neural system, induced synchronization to weak, periodic signals corresponding to the healthy condition at appropriate feedback strength, although this synchronization was restricted to a relatively low frequency of the input signal. Thus, the chaotic resonance induced by the RRO feedback signal facilitates the transition to a state that is close to healthy, periodic frontal neural activity in the case where this activity has a relatively low frequency. Additionally, the combined amount of perturbation due to the RRO feedback signal and the periodic input signal was significantly smaller than that required for inducing a synchronous state by applying only the periodic signal.

First, we must consider the reason the RRO feedback signal facilitates synchronization by small perturbations. Over the past few decades, studies of non-linear control aimed at stabilizing chaotic activity have proposed many methods such as the Ott-Grebogi-Yorke method, the delay feedback method, and the *H*_∞_ method (Ott et al., 1990; Pyragas, 1992; Nakajima, 1997; Jiang et al., 2005; reviewed in Schöll and Schuster, 2008). These conventional chaos control methods stabilize the chaotic orbit to equilibrium points and periodic orbits. In contrast, the RRO feedback method does not eliminate chaotic behavior but adjusts local maximum and minimum values of the map function; consequently, the feedback strength at which chaotic behavior is maintained in RRO is smaller than that of conventional chaos control methods, in which chaotic behavior is completely suppressed (Nobukawa et al., 2018b). Moreover, by virtue of chaotic resonance at around the attractor-merging bifurcation induced by the RRO feedback signal, chaos-chaos intermittency synchronizes with input signals even at low input-signal strength (Sinha, 1999; Nishimura et al., 2000; reviewed in Anishchenko et al., 2007; Rajasekar and Sanjuán, 2016). Utilizing these advantages of the RRO feedback method and of chaotic resonance should facilitate the transition of the disturbed neural activity of BD to a healthy periodic state.

Furthermore, the application of RRO feedback signals with periodic input signals shows great promise for actual chronotherapy practice. In current chronotherapy, the administration of the light stimulus and the medication occurs at a fixed time each day to enable the transition of neural activity to a periodic state with a circadian period (Yeragani et al., 2003; Glenn et al., 2006; Bonsall et al., 2011; Moore et al., 2014; reviewed in Albrecht, 2013). This treatment may correspond to the case we consider here, in which neural activity is stabilized by applying only a periodic input signal (see Figure 10). The application of the light stimulus and medication on a schedule modulated by the daily frontal neural activity of each patient would correspond to the application of RRO feedback signals in combination with the periodic input signal, in which the amount of perturbation needed for the transition to the periodic state is expected to be significantly reduced. That is, this strategy may lead to a reduction in the amounts of stimulus and medication necessary to transition from a disturbed frontal neural activity to a healthy periodic state. Furthermore, this effect might also contribute to a reduction in mixed states, hypomania, and autonomic hyperactivations that can occur in BD chronotherapy due to overapplication of light stimuli and medication. Additionally, methods for measuring the daily variation of frontal neural activity are now under development, with a focus on EEG approaches (Croce et al., 2018; González et al., 2019). These methods might contribute to the realization of a form of chronotherapy modulated by RRO feedback.

The following limitations of this study must be considered. First, only the neural system composed of frontal and sensory cortices was considered. However, the circadian rhythms targeted in chronotherapy are produced not only by the frontal and sensory cortices but also by the hypothalamus (Hadaeghi et al., 2016; Bayani et al., 2017). Therefore, the evaluation of chaotic resonance induced by the RRO feedback method in a neural system comprising both the frontal/sensory cortex and the hypothalamus is important for evaluating its applicability to chronotherapy. Second, we used competition between excitatory and inhibitory neurons in this study to describe long-term neural dynamics in the frontal cortex. However, the questions of what reflects the long-term dynamics of brain activity and what mechanism produces it are currently controversial (Croce et al., 2018; González et al., 2019). Therefore, it is important that our proposed method be verified with models based on other neural mechanisms for producing long-term neural dynamics in the frontal cortex. In such evaluations, the use of spiking neuron models, which exhibit highly realistic neurodynamics (Nobukawa et al., 2017, 2018a, reviewed in Ma and Tang, 2017), would enhance the pathological validity of the neural network used to simulate BD (Brambilla et al., 2003; Hasler et al., 2007; Sanacora et al., 2012; Schloesser et al., 2012; reviewed in Chiapponi et al., 2016). Third, from the viewpoint of chaotic resonance, the disturbed neural activity described as chaotic dynamics in this study was close to the healthy periodic state. However, to stabilize more challenging forms of chaotic behavior, other candidate chaos control methods must be considered; we plan to research these points in the future. In addition to model-based studies, the methods of measuring the daily-timescale variation in the frontal neural activity that have recently been proposed (Croce et al., 2018; González et al., 2019) will be crucial for applications and will aid in the estimation of the controlling parameters required by RRO feedback methods.

In conclusion, in this simulation study, chaotic resonance induced by the RRO feedback method enabled the disturbed frontal neural activity characteristic of BD to be transitioned close to a healthy periodic state by relatively weak perturbations. Despite its limitations, this study demonstrated that chronotherapy modulated by the RRO feedback method might be a new type of minimally invasive therapy for BD.

## Data Availability Statement

The raw data supporting the conclusions of this article will be made available by the authors, without undue reservation.

## Author Contributions

SN, HN, and TT conceived the methods. SN and NW analyzed the results, wrote the main text, and prepared all figures. SN and HD conducted the experiments. All authors have reviewed the manuscript.

## Conflict of Interest

The authors declare that the research was conducted in the absence of any commercial or financial relationships that could be construed as a potential conflict of interest.
